# Human and machine recognition of dynamic and static facial expressions: prototypicality, ambiguity, and complexity

**DOI:** 10.3389/fpsyg.2023.1221081

**Published:** 2023-09-12

**Authors:** Hyunwoo Kim, Dennis Küster, Jeffrey M. Girard, Eva G. Krumhuber

**Affiliations:** ^1^Departmet of Experimental Psychology, University College London, London, United Kingdom; ^2^Cognitive Systems Lab, Department of Mathematics and Computer Science, University of Bremen, Bremen, Germany; ^3^Department of Psychology, University of Kansas, Lawrence, KS, United States

**Keywords:** emotion facial expression, dynamic, movement, prototypicality, ambiguity

## Abstract

A growing body of research suggests that movement aids facial expression recognition. However, less is known about the conditions under which the dynamic advantage occurs. The aim of this research was to test emotion recognition in static and dynamic facial expressions, thereby exploring the role of three featural parameters (prototypicality, ambiguity, and complexity) in human and machine analysis. In two studies, facial expression videos and corresponding images depicting the peak of the target and non-target emotion were presented to human observers and the machine classifier (FACET). Results revealed higher recognition rates for dynamic stimuli compared to non-target images. Such benefit disappeared in the context of target-emotion images which were similarly well (or even better) recognised than videos, and more prototypical, less ambiguous, and more complex in appearance than non-target images. While prototypicality and ambiguity exerted more predictive power in machine performance, complexity was more indicative of human emotion recognition. Interestingly, recognition performance by the machine was found to be superior to humans for both target and non-target images. Together, the findings point towards a compensatory role of dynamic information, particularly when static-based stimuli lack relevant features of the target emotion. Implications for research using automatic facial expression analysis (AFEA) are discussed.

## 1. Introduction

Much of our understanding of facial expressions of emotions has come from studies of static displays typically captured at their peak (Dawel et al., 2022). Static expressions have the advantage that they can be strictly controlled, allowing observers to focus on the key features of interest. Not surprisingly, static images have been widely used in studies exploring the recognition of the basic six emotions (Calvo and Nummenmaa, 2016; Barrett et al., 2019). Due to their lower ecological validity, however, the last two decades have seen increased questioning and criticism of this type of stimulus. Given that facial expressions evolve over time, they are intrinsically dynamic events. Accordingly, facial movement has been shown to aid expression recognition (e.g., Wehrle et al., 2000; Ambadar et al., 2005; Cunningham and Wallraven, 2009) and facilitate the extraction of emotion-relevant content from faces (for reviews, see Lander et al., 1999; Krumhuber et al., 2013, 2023; Krumhuber and Skora, 2016; Dobs et al., 2018), such as expression authenticity (Krumhuber et al., 2013; Zloteanu et al., 2018), naturalness (Sato and Yoshikawa, 2004) and intensity (Biele and Grabowska, 2006; Widen and Russell, 2015). Nonetheless, the effects of movement are not uncontested, with some studies showing little or no benefits of dynamic information (e.g., Knight and Johnston, 1997; Lander et al., 1999; Kamachi et al., 2001; Fiorentini and Viviani, 2011; Gold et al., 2013). The present research aims to compare static versus dynamic expressions in human and machine analysis, thereby exploring the role of featural parameters in emotion recognition.

Despite substantial evidence showing a dynamic advantage, several studies have failed to find the respective benefits of movement. For example, the advantage was found to disappear when identification was already close to perfect, with static stimuli that were highly distinctive in expression (Kamachi et al., 2001 experiment 2; Kätsyri and Sams, 2008; Gold et al., 2013). Also, the effect of movement diminished for static displays presented for more than 1,000 ms, which naturally allows for a deeper exploration of the facial stimulus (Bould and Morris, 2008; Kätsyri and Sams, 2008). Finally, movement of the face may not always be necessary for non-degraded or full-intensity expressions (Ambadar et al., 2005; Bould and Morris, 2008; Tobin et al., 2016; Blais et al., 2017). In those cases, static snapshots can be sufficient to recognise emotions. Such counterevidence aligns with arguments proposing a compensatory role of dynamic information, particularly when static cues are inaccessible or insufficient (Ehrlich et al., 2000; Wehrle et al., 2000; Atkinson et al., 2004; Ambadar et al., 2005). For example, dynamic expressions aid the recognition of degraded or distorted stimuli such as in point-light displays, synthetic displays, or shuffled morphed sequences (e.g., Wallraven et al., 2008; Cunningham and Wallraven, 2009; Dobs et al., 2018; Plouffe-Demers et al., 2019). Similarly, facial movement facilitates the recognition of weakly expressed and non-basic emotions (guilt, shame), which may be more subtle and nuanced in their appearance (Ambadar et al., 2005; Bould and Morris, 2008; Cassidy et al., 2015; Yitzhak et al., 2022).

While attempts have been made to specify the conditions under which the dynamic advantage occurs, it is still unclear when dynamic information matters and when it does not. In most past studies, static displays were used to depict the peak of the target emotion (Harwood et al., 1999; Kamachi et al., 2001; Bould and Morris, 2008; Gold et al., 2013). Such high-intensity features, with their specific shapes and spatial arrangement, may leave little scope for the additional benefits offered by movement. The present research is the first to compare dynamic expressions with static images extracted from various time points of the facial display. In particular, we explore whether peak frames of the target emotion (e.g., the image frame with the highest surprise evidence within a surprise video; see Dente et al., 2017) achieve recognition rates that are similar to dynamic stimuli (e.g., a full-length surprise video) and higher compared to those of non-target emotions (e.g., image frames with the highest anger, fear, disgust, happiness or sadness evidence within a surprise video).

Beyond this comparison of dynamic expressions to automatically extracted single images, the present work examines three key featural parameters and their contribution to emotion recognition. According to Basic Emotion Theory (BET), a small number of fundamental emotions are characterised by *prototypical* patterns of facial actions (Ekman, 1982, 1992). That is, when an emotion is elicited a particular set of action units is triggered by specific muscular movements (Ekman et al., 2002). These unique configurations of prototypical facial displays offer a quick and accurate feature-based categorisation of expressions as they are unambiguously linked with discrete emotion categories (see Ekman, 2003; Calvo and Nummenmaa, 2016). Such categorical distinctiveness makes them perceptually salient, thereby providing a shortcut to emotion recognition (Calvo and Fernández-Martín, 2013). Hence, facial displays closely resembling those prototypes are more easily and rapidly classified (Young et al., 1997; Matsumoto et al., 2009; Matsumoto and Hwang, 2014). Conversely, accuracy is thought to drop for non-prototypical expressions (Wagner et al., 1986; Motley and Camden, 1988; Naab and Russell, 2007; Barrett et al., 2019).

While prototypicality crucially functions as a perceptual indicator of emotion category, most of the facial expressions seen in everyday life are likely to be ambiguous, fractional, and/or blended (Scherer and Ellgring, 2007; Calvo et al., 2014). That is, they often convey a mixture of emotions (Halberstadt et al., 2009; Hassin et al., 2013; Parkinson, 2013) or partial versions of configurations, with a great amount of idiosyncrasy and variability beyond uniform configurations of a single emotion (Du et al., 2014; Du and Martinez, 2015). To capture these deviations, it is therefore important to define a second featural parameter.

*Ambiguity* arises when an expression displays multiple basic emotions (i.e., when facial expressions are categorically ambiguous), thereby containing contradictory emotional information. Given that classification decisions typically rely on the most distinctive facial features (Fiorentini and Viviani, 2009; Calvo et al., 2012; Tanaka et al., 2012; Du et al., 2014), ambiguous expressions are often subject to misclassification and interpretation biases (Calvo et al., 2012; Ito et al., 2017; Kinchella and Guo, 2021). In turn, recognition accuracy is reduced (Calder et al., 2000b; Neta and Whalen, 2010) because people are perceptually less able to identify several emotions at once (Ito et al., 2017; Kinchella and Guo, 2021). Neuroscientific evidence points towards the role of the amygdala, which encodes not only the intensity but also the categorical ambiguity of an expression (Ito et al., 2017). Since the processing of ambiguous displays requires more cognitive effort, confidence ratings tend to be lower and reaction times are prolonged (Calvo et al., 2012; Wang et al., 2017).

Notwithstanding its importance, empirical evidence regarding expression ambiguity remains elusive mainly due to the lack of a common metric. While some studies define it as the degree of closeness to categorical boundaries (Halberstadt et al., 2009; Wang et al., 2017; Kinchella and Guo, 2021), others conceptualise it as the omission of core emotional cues (Matsumoto and Hwang, 2014). This could be problematic as both definitions indicate different expression characteristics. Additionally, most prior research has manipulated (rather than measured) ambiguity by creating blended, morphed, or composite face stimuli (Nummenmaa, 1988; Calder et al., 2000a,b). Such an approach may result in unnaturalistic displays which are not representative of the type of expressions seen in real-life situations. The present work therefore introduces a new ambiguity measure that is based on the perceived presence of two or more emotions.

Finally, expression *intensity* has been consistently shown to influence emotion recognition. Specifically, intense displays enhance accurate classification and response times (e.g., Young et al., 1997; Matsumoto, 1999; Matsumoto et al., 2002; Palermo and Coltheart, 2004; Ambadar et al., 2005; Jones et al., 2018). Also, they lead to higher intensity and confidence ratings (Calder et al., 2000a; Recio et al., 2013), as well as agreement ratings between viewers (Matsumoto et al., 2002; Matsumoto and Hwang, 2014). In contrast, weak expressions tend to be less accurately categorised (although above chance level, Matsumoto and Hwang, 2014) and are subject to greater confusion and uncertainty in emotion judgements (Matsumoto et al., 2002; Bould and Morris, 2008; Ichikawa et al., 2014).

The intensity of expressions may play a crucial role in detecting individual facial configurations because intense expressions often contain diagnostic features of facial prototypes. Expression prototypicality is therefore likely to co-occur with higher expressive intensity. Only a few studies to date have tried to identify their relative influence, suggesting that prototypicality is a more important feature for emotion classification than intensity (Matsumoto et al., 2002; Matsumoto and Hwang, 2014). Nonetheless, both parameters are likely to be confounded as expression intensity usually concerns emotion-relevant facial actions such as those predicted by BET. This makes intensity not representative of the overall expressivity of the face, but of the degree of emotion in a facial expression. More intense emotional expressions (especially when they are posed) are likely to be more prototypical and vice versa. In order to conceptualise expression intensity as a measure that is independent from its emotional connotation, we therefore introduce a new metric called “complexity” which captures the intensity of all action units in the face.

While traditional measures of intensity consider the strength of Action Units (AUs) contractions, our measure of “complexity” quantifies the number of contracting AUs, irrespective of their individual intensities. This approach captures the richness of facial actions without being influenced by the strength of individual AU contractions. Although the probabilities of AU-occurrences may correlate with their respective intensities, complexity provides a comprehensive representation of facial expressivity. This distinction is crucial as facial expressions often involve a mixture of AUs and may not strictly adhere to the prototypical expressions of basic emotions. As such, our measure of complexity offers a unique perspective that is distinct from traditional measures of intensity, which are typically tied to the intensity of emotion-specific AUs.

Quantifying featural parameters necessitates an objective classification of facial expressions, which is a time-consuming and resource-intensive process for human coders (De la Torre and Cohn, 2011). With rapid advances in the field of affective computing, commercial and open-source algorithms for automated facial expression analysis (AFEA) are now widely available (Cohn and Sayette, 2010). These can reliably classify discrete emotions as well as facial actions (Littlewort et al., 2011; Lewinski et al., 2014). Given that most classifiers have been trained based on the theoretical principle proposed by the Facial Action Coding System (FACS, Ekman et al., 2002; Calvo et al., 2018), recognition performance is found to be comparable to human coders (Skiendziel et al., 2019; Krumhuber et al., 2021a) and other physiological measurements (Kulke et al., 2020; Höfling et al., 2021), sometimes even outperforming human raters (Krumhuber et al., 2021b). In most cases, the distinctive appearance of highly standardised expressions benefits the featural analysis by machines (Pantic and Bartlett, 2007).

Despite several attempts to validate AFEA, its performance on non-prototypical, subtle, and dynamic expressions needs further attention, with studies showing substantial variation in recognition success. For example, hit rates drop remarkably when an expression moves farther away from basic emotion prototypes (Stöckli et al., 2018; Küntzler et al., 2021). Likewise, machines frequently misclassify expressions that are weak in intensity (Calvo et al., 2018; Küntzler et al., 2021), resulting in recognition rates often lower than those of humans (Mandal et al., 2015; Yitzhak et al., 2017). Since machines rely heavily on physical features of an expression (Del Líbano et al., 2018), less prototypical and more subtle displays of emotion pose a greater challenge for AFEA (Calvo et al., 2018). This is particularly evident for dynamic expressions, which often include large segments of frames with comparatively subtle features. In consequence, machine accuracy has been shown to drop for dynamic compared to static stimuli commonly taken at the peak of the emotional display (Stöckli et al., 2018; Skiendziel et al., 2019; Onal Ertugrul et al., 2023). To date, the role of dynamic information in AFEA is still poorly understood, with performance varying substantially across stimulus conditions (Yitzhak et al., 2017; Dupré et al., 2019; Krumhuber et al., 2021b).

There is suggestive albeit ambivalent evidence for the dynamic advantage with inconclusive findings on why and when facial movements offer benefits for recognition. The present research aims to fill this knowledge gap by investigating the conditions under which dynamic information exerts its facilitative effects on emotion classification. It does so by comparing dynamic stimuli with static peak images that show either the target or non-target emotion (thereafter referred to as “target-images” and “non-target images”). In line with previous research on the dynamic advantage (Wehrle et al., 2000; Ambadar et al., 2005; Cunningham and Wallraven, 2009), we predicted superior recognition rates for dynamic displays when compared to static (non-target) images consisting of peak frames that are unreflective of the target emotion. In other words, images taken from any time point of the expression may show minimal benefits, resulting in recognition rates lower than those of dynamic expressions. However, the opposite pattern was expected for static images showing the peak frame of the target emotion (target-images). Given that these are highly distinctive and intense displays of the relevant emotion (Kamachi et al., 2001; Kätsyri and Sams, 2008; Gold et al., 2013), they should be easier to recognise, with performance rates exceeding those of dynamic expressions. To investigate what makes the expression recognisable, we tested the relative contribution of three featural parameters – prototypicality, ambiguity, and complexity – to emotion recognition. If the stimuli closely resemble discrete emotion categories as proposed by BET, they should be more prototypical and intense as well as less ambiguous in appearance (Neta and Whalen, 2010; Matsumoto and Hwang, 2014; Jones et al., 2018). Stimuli that show well-recognisable discrete emotions should also be more complex than most other patterns of facial actions. Furthermore, prototypicality and ambiguity as its counterpart should predict emotion recognition, particularly in machines which have often been trained on posed/acted datasets (Pantic and Bartlett, 2007), making them potentially superior to human observers in classification accuracy (Krumhuber et al., 2021b).

Two studies were conducted to test the above hypotheses. Study 1 focused on AFEA to compare video (dynamic), target and non-target images (static), and define measures of prototypicality, ambiguity, and complexity. As a way of validating the machine data, we also obtained ratings from human observers on target and non-target images. Study 2 focused on human observers with the aim to replicate the findings from the first study with a subset of the stimuli and a larger sample of participants.

## 2. Experiment 1

The first study aimed to test for the dynamic advantage in AFEA, thereby comparing recognition rates of video (dynamic), target and non-target images (static). Human observer ratings were also obtained for target and non-target images as a source of machine validation. In addition, we explored the relative contribution of prototypicality, ambiguity, and complexity to image and video recognition, and whether video recognition can be predicted based on six images that represent the respective peak expressions for the basic emotions.

### 2.1. Method

#### 2.1.1. Stimulus material

162 facial expression videos portraying the six basic emotions (anger, disgust, fear, happiness, sadness, and surprise) were obtained from Krumhuber et al. (2021b). Stimuli originated from a range of databases showcasing a mixture of emotion elicitation procedures (e.g., instruction to perform an expression, scenario enactment, emotion-eliciting tasks). For each video, machine analysis was performed using a commercial software called FACET (Littlewort et al., 2011), which provides estimates for facial expressions of the six basic emotions (anger, disgust, fear, happiness, sadness, surprise) and 20 Action Units (AU1, 2, 4, 5, 6, 7, 9, 10, 12, 14, 15, 17, 18, 20, 23, 24, 25, 26, 28, and 43; Ekman et al., 2002). It outputs evidence scores on a frame-by-frame basis, estimating the likelihood that a human observer would code the frame as containing each emotion and action unit. Evidence values are shown on a decimal logarithmic scale centred around zero, with zero indicating 50% probability, negative values indicating that an expression is likely not present, and positive values indicating that an expression is likely to be present (Dente et al., 2017).

**Figure 1:**
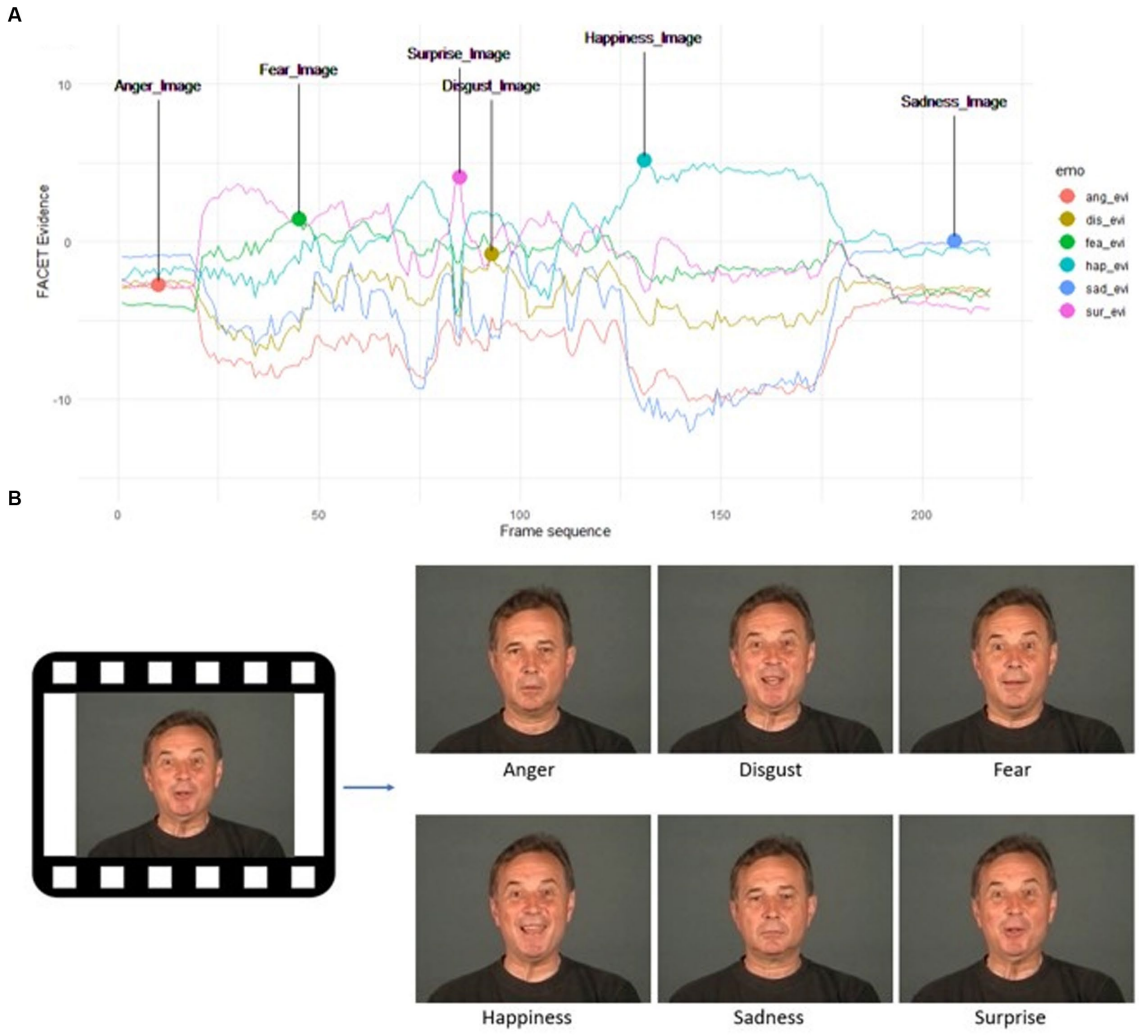
Example of image selection procedure, showing the highest FACET evidence values for each of the six basic emotions as extracted from a surprise video **(A)**. The surprise image (bottom right) is the target image for the surprise video (as labelled by the dataset authors), whereas the other five mages are non-target images **(B)**. Reproduced with permission from (Nadia Mana / Battocchi et al., 2005).

Within each video, six frames with the highest individual evidence value for the six basic emotions were identified based on the raw FACET output. Extractions were performed automatically via Python and FFmpeg. Among the six frames, one image was indicative of the “target” emotion (e.g., the frame with the highest surprise evidence score from a video that was labelled by the dataset authors as surprise), and five images were indicative of “non-target” emotions (e.g., frames with the highest anger, disgust, fear, happiness, and sadness evidence scores from a surprise video; see Figure 1). To this end, a total of 972 static facial images (162 videos × 6 images) were extracted. The number of portrayals was equally balanced across disgust, fear, happiness, and surprise (168 images each), except for anger (144 images) and sadness (156 images) which had fewer portrayals because they were not available in some of the databases. All image stimuli were rendered in colour and had an approximate resolution of 550 × 440 pixels.

To achieve comparability with the confidence ratings provided by human observers, the raw FACET evidence values for each of the six basic emotions and 20 AUs were initially converted into probabilities by using the formula provided in the FACET documentation (iMotions, 2016) and then into confidence odds scores (for a similar procedure see Krumhuber et al., 2021a). Let $x_{ijk}$ represent the evidence value for emotion or AU k in image j from video i . This value can be converted into probability $\left(p_{ijk}\right)$ and odds $\left(o_{ijk}\right)$ units using Eqs. 1, 2, respectively:


(1) $$p_{ijk}=\frac{1}{1+10^{-x_{ijk}}}$$

(2) $$\begin{array}{c} o_{ijk}=\frac{1}{1/p_{ijk}-1} \end{array}$$

#### 2.1.2. Human observers

##### 2.1.2.1. Participants

One hundred and fifty-four participants (76 females), aged between 18–60 years (*M* = 29.78, *SD* = 11.85), volunteered to take part in the study. Participants were recruited face-to-face or online via the departmental subject pool and Prolific Academic’s digital recruitment platform. Participants received course credits or £10 for taking part in the study. All participants were White/Caucasian and identified as British or European and ordinary residents in the UK. Ethical approval was granted by the Department of Experimental Psychology at University College London, United Kingdom.

##### 2.1.2.2. Procedure

To reduce participation time, a subset of 162 facial images portraying the six basic emotions were extracted from the 972 static expression stimuli and were randomly presented. As such, every participant viewed one image from each video. The number of portrayals was balanced across the six emotions. Each facial expression was presented for 15 s using the Qualtrics software (Provo, UT). For each facial stimulus, participants rated the extent (from 0 to 100%) to which each of the six emotions (anger, disgust, fear, happiness, sadness, and surprise) is recognisably expressed in the face. At least one emotion rating per image (greater than 1% for any emotion) had to be given. Participants could respond using multiple sliders (if applicable) to choose the exact confidence levels for each response category.

#### 2.1.3. Parameters

##### 2.1.3.1. Prototypicality

We defined expression “prototypicality” as the degree to which the combination of AUs estimated to be present in a facial expression matches the prototypical facial expression configuration proposed by Basic Emotion Theory (Ekman, 1992). The FACS manual (Ekman et al., 2002) was used to define the full prototype and major variants of each basic emotion. The odds of FACET AU scores for the target emotion were summed up and weighted by a factor of 1 (full prototype, e.g., AU1 + 2 + 5 + 26 for surprise) or 0.75 (major variant, e.g., AU1 + 2 + 5 for surprise). This resulted in an estimated prototypicality score for each image, with higher scores indicating greater prototypicality of the expressed emotion (for a similar procedure, see Krumhuber et al., 2021a). Prototypicality for emotion k in image j from video i was calculated as:


(3) $$\begin{array}{c} PRO_{ijk}=\overset{v}{\underset{l=1}{\sum }}O_{ijkl}w_{kl} \end{array}$$

where *O_ijkl_* is the FACET-estimated odds that image j from video i contains prototype l from emotion k and $w_{kl}$ is the weight of prototype l from emotion k (i.e., 1 if a full prototype and 0.75 if a major variant). To calculate the prototypicality for emotion k in video i (across all m images), we averaged the prototypicality for that emotion across all m images (i.e., m=6 ).


(4) $$\begin{array}{c} PRO_{ik}=\frac{1}{m}\overset{m}{\underset{j=1}{\sum }}PRO_{ijk} \end{array}$$

##### 2.1.3.2. Ambiguity

We defined expression “ambiguity” as the degree to which the facial expression is classified as containing multiple basic emotions, which makes the expression categorically unclear (Kinchella and Guo, 2021). To this end, we used normalised entropy as a metric to represent the amount of uncertainty in emotion classification for each image (Shannon, 1948). Entropy is high when multiple emotions have high estimated probabilities and low when only a single emotion has a high estimated probability. The ambiguity of image j from video i (in terms of the q different emotions) was calculated using the following equation:


(5) $$\begin{array}{c} AMB_{ij}=-\sum_{k=1}^{q}\frac{p_{ijk}}{\log\left(q\right)} \end{array}$$

where $p_{ijk}$ is the FACET-estimated probability that image j from video i contains emotion k . (Note that the logarithm bases do not matter due to their division). To calculate the ambiguity for video i (across all m images), we averaged the ambiguity across all m images (i.e., m=6 ).


(6) $$\begin{array}{c} AMB_{i}=\frac{1}{m}\overset{m}{\underset{j=1}{\sum }}AMB_{ij} \end{array}$$

##### 2.1.3.3. Complexity

We defined expression “complexity” as the average estimated probability across all 20 FACET AU estimates in each image. This resulted in an estimated complexity score for each image, with higher scores indicating more complex expressions (with evidence of more AUs present). This complexity measure therefore differs from other conceptualisations of “intensity” by taking all FACET AUs into account and using their probability of occurrence rather than their estimated intensity. The complexity for image j from video i was calculated as:


(7) $$\begin{array}{c} COM_{ij}=\frac{1}{m}\overset{f}{\underset{l=1}{\sum }}P_{ijl} \end{array}$$

where $p_{ijl}$ is the FACET-estimated probability that image j from video i contains AU l and f=20 (i.e., the superset of all estimated AUs). To calculate the complexity for video i (across all m images), we averaged the complexity across all m images (i.e., m=6 ).


(8) $$\begin{array}{c} COM_{i}=\frac{1}{m}\overset{m}{\underset{j=1}{\sum }}COM_{ij} \end{array}$$

#### 2.1.4. Data preparation

FACET recognition accuracy for both video and image was calculated by determining whether the emotion with the highest recognition score matched the target emotion label given by the database authors. As FACET is an algorithm-based classifier that provides the same values across trials, recognition accuracy was binary in the form of either 0 (incorrect) or 1 (correct). To compare FACET and human performance, the recognition scores by human observers were also converted into this binary format as a function of whether the majority (> 50%) of participants correctly recognised the target emotion.

### 2.2. Results

#### 2.2.1. 6-images as predictor of video recognition

We first tested whether emotion classification accuracy of the video can be predicted from the recognition of the 6 extracted images. For this, a multilevel logistic regression model predicting video-level emotion classification accuracy (by FACET) was estimated with a random intercept for each video and fixed slope for the sum of correct image-level emotion classification accuracy (per video). The results revealed a significant main effect (exp(β) = 2.86, Wald = 35.63, *p* < 0.001, exp(95%CI) [2.10, 4.22]), indicating that the odds of correct video-level emotion classification increased by 186% for each additional correct image-level emotion classification.

#### 2.2.2. Video vs. target image vs. non-target images

**Figure 2:**
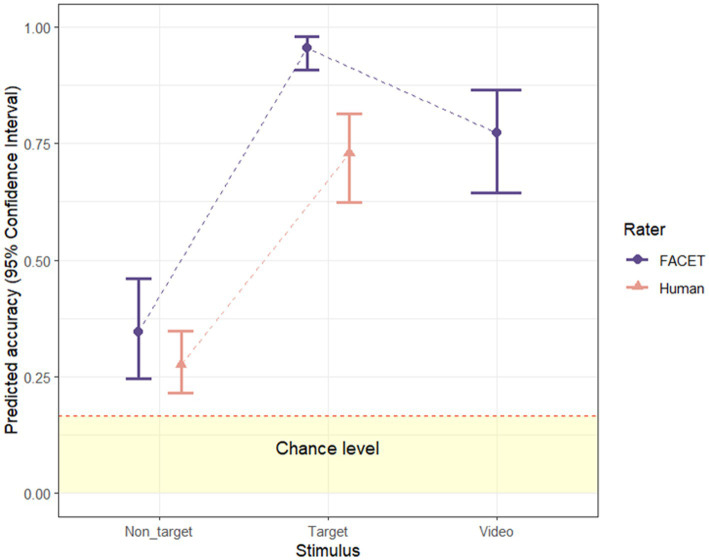
FACET and human recognition accuracy for video, target- and non-target images. Error bars represent upper and lower bounds of 95% confidence interval. Dashed red line indicates 1/6 conservative chance level (Krumhuber et al., 2021b).

To examine whether recognition accuracy differs as a function of stimulus type (video vs. target image vs. non-target images), a multilevel logistic regression analysis with a random intercept by video was conducted on the FACET accuracy data. The odds of correct emotion classification were significantly higher for target images than for non-target images (exp(β) = 40.66, Wald = 99.48, *p* < 0.001, exp(95%CI) [20.40, 88.10]) and were significantly higher for the video (exp(β) = 6.47, Wald = 48.37, *p* < 0.001, exp(95%CI) [3.87, 11.12]) than for non-target images (see Figure 2). Interestingly, the odds of correct emotion classification were significantly lower for the video than for target images (exp(β) = 0.16, Wald = 21.98, *p* < 0.001, exp(95%CI) [0.07, 0.34]). As such, the dynamic advantage only occurred for non-target images, but not target images. Overall, recognition accuracy was highest for the target image, followed by the video and non-target images (see Figure 2).

We conducted another multilevel logistic regression analysis with stimulus type (target vs. non-target images) and rater type (FACET vs. human observers) as predictors and with a random intercept for each video. The results revealed significant main effects of stimulus type, (exp(β) = 7.05, Wald = 74.47, *p* < 0.001 exp(95%CI) [4.52, 10.98]) and rater type (exp(β) = 1.65, Wald = 16.16, *p* < 0.001 exp(95%CI) [1.29, 2.11]), as well as a significant interaction between the two (exp(β) = 2.38, Wald = 6.23, *p* = 0.035 95%CI [1.20, 4.70]). For both FACET and humans, target images were better recognised than non-target images (*ps* < 0.001). Thus, the target peak image seemed to be a better exemplar of the expression in human and machine analysis. Results also revealed that recognition accuracy of FACET was significantly higher than that of humans for both target and non-target images (*ps* < 0.001).

#### 2.2.3. Prototypicality, ambiguity, and complexity of expression

To investigate what makes the expression recognisable, separate Welch’s *t*-tests were conducted to compare stimulus types (target vs. non-target images) in terms of prototypicality, ambiguity, and complexity. As expected, target images were significantly more prototypical (*M_target_* = 64.08, *SD* = 34.11 vs. *M_non-target_* = 37.18, *SD* = 33.16), *t*(226.03) = 9.21, *p* < 0.001, *d* = 0.81, less ambiguous (*M*_t*arget*_ = 29.79, *SD* = 25.60 vs. *M_non-target_* = 46.99, *SD* = 22.20), *t*(212.16) = 5.18, *p* < 0.001, *d* = 0.75, and more complex (*M_target_* = 28.22, *SD* = 7.76 vs. *M_non-target_* = 24.60, *SD* = 9.72), *t*(272.75) = 5.18, *p* < 0.001, *d* = 0.38, than non-target images.

**Figure 3:**
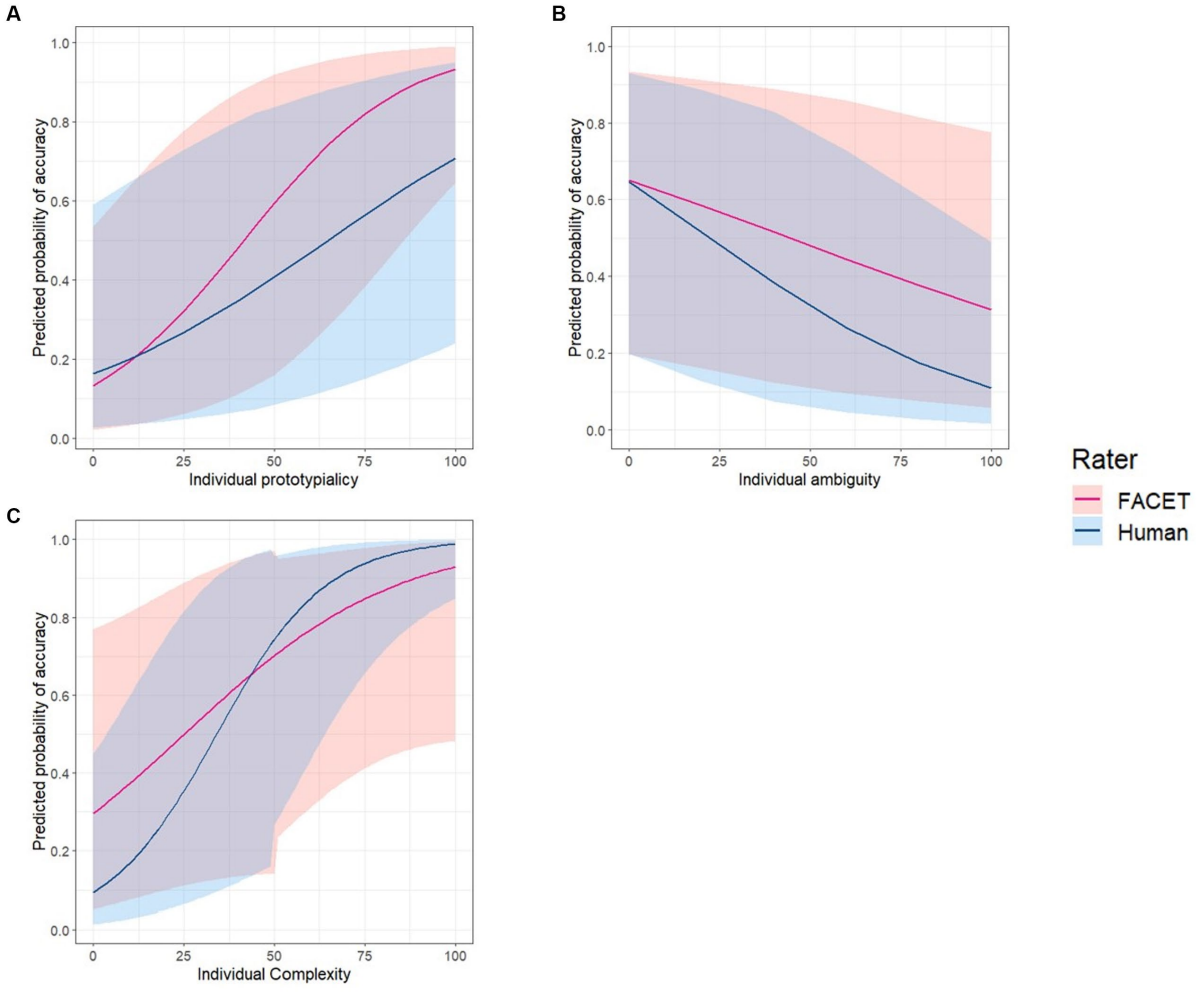
Predicted power of prototypicality, ambiguity, and complexity for image recognition accuracy in FACET and humans. Regression line indicates the relationship between image recognition accuracy (red: FACET, blue: Human) and individual scores of **(A)** prototypicality, **(B)** ambiguity, and **(C)** complexity. The line shades represent upper and lower bounds 95% confidence interval at each predictor score point.

Next, we examined the relative contribution of each parameter to emotion classification accuracy. For this, a multilevel logistic regression model predicting each image’s classification accuracy was estimated with random intercepts for each video and fixed slopes for prototypicality, ambiguity, complexity, rater type, and the interaction of rater type with the other three measures. Results revealed a significant main effect of prototypicality (exp(β) = 1.05, Wald = 135.06, *p* < 0.001, exp(95%CI) [1.04, 1.05]), ambiguity (exp(β) = 0.99, Wald = 9.63, *p* = 0.002, exp(95%CI) [0.98, 0.99]), and complexity (exp(β) = 1.04, Wald = 8.36, *p* = 0.004, exp(95%CI) [1.01, 1.06]). All three parameters showed a significant interaction effect with rater type (*ps* < 0.01). Post-hoc tests revealed that the effects of prototypicality (exp(β) = 1.02, Wald = 32.14, *p* < 0.001, exp(95%CI) [1.01, 1.03]) and ambiguity (exp(β) = 1.01, Wald = 7.90, *p* = 0.005, exp(95%CI) [1.00, 1.02]) were significantly greater for FACET than for humans. In contrast, the effect of complexity (exp(β) = 1.03, Wald = 10.91, *p* < 0.001, exp(95%CI) [0.95, 0.99]) was significantly greater for humans than FACET (see Figure 3 and Table 1).

**Table 1:** Model estimates for FACET and human image recognition accuracy, showing main and interaction effect estimates in logits, upper and lower bounds of exponentiated 95% confidence intervals, and significance of each predictor (Study 1).

Predictor	exp(β)	Wald	L95%CI	H95%CI	*p*
Prototypicality	1.05	135.06	1.04	1.05	>0.001***
Ambiguity	0.99	9.63	0.98	0.99	0.002**
Complexity	1.04	8.36	1.01	1.06	0.004**
Prototypicality:Rater	0.99	23.06	0.98	0.99	>0.001***
Ambiguity:Rater	0.99	6.70	0.98	1.00	0.010*
Complexity:Rater	1.02	8.68	1.01	1.04	0.003**

Finally, we explored the partial association of each parameter with video-level recognition accuracy. For this, a multilevel logistic regression model predicting video-level emotion classification accuracy (by FACET) was estimated with random intercepts for each source database and fixed slopes for video-level prototypicality, ambiguity, and complexity. Results revealed a significant main effect of prototypicality (exp(β) = 1.01, Wald = 7.54, *p* = 0.006, exp(95%CI) [1.00, 1.02]), and ambiguity (exp(β) = 0.97, Wald = 26.12, *p* < 0.001, exp(95%CI) [0.96, 0.98]). The main effect of complexity was marginally significant (exp(β) = 0.98, Wald = 3.81, *p* = 0.051, exp(95%CI) [0.95, 1.00]). In general, the odds of recognition accuracy increased by 1% for each unit increase in prototypicality, while it decreased by 3% for each unit increase in ambiguity (see Table 2).

**Table 2:** Model estimates for FACET video recognition accuracy, showing main effect estimates in logits, upper and lower bounds of exponentiated 95% confidence intervals, and significance of each predictor (Study 1).

Predictor	exp(β)	Wald	L95%CI	H95%CI	*p*
Prototypicality	1.01	7.54	1.00	1.02	0.006**
Ambiguity	0.97	26.12	0.96	0.98	>0.001***
Complexity	0.98	3.81	0.95	1.00	0.051

### 2.3. Discussion

The results of the first study demonstrated considerable variation in recognition accuracy as a function of stimulus type. On average, recognition accuracy was highest for target images, followed by the video and non-target images. In accordance with previous findings (Harwood et al., 1999; Gepner et al., 2001; Ambadar et al., 2005; Bould and Morris, 2008), movement (in the form of videos) aided emotion classification over non-target images that were generally less prototypical and complex but more ambiguous than target images. Such a dynamic advantage was absent in comparison to static images which showed the expression at its peak intensity of the target emotion. Additionally, accurate recognition of the video was successfully predicted by the six images, pointing towards the usefulness of single images in video prediction.

Regarding featural parameters, higher prototypicality and complexity but lower ambiguity encouraged correct recognition in both humans and the machine. While prototypicality and ambiguity were better predictors of machine performance, complexity (as a reflection of overall expressivity) was more effective in predicting human accuracy. These findings are in line with prior works suggesting that AFEA relies heavily on specific facial configurations (Zeng et al., 2009; Krumhuber et al., 2021a) due to its training on a few – often posed/acted – datasets (Pantic and Bartlett, 2007) while humans tend to process expressions more holistically including all facial actions (Calvo et al., 2012). When comparing human and machine performance, a similar pattern was observed in the sense that accuracy decreased for non-target (*vs* target) images. Interestingly, the machine outperformed humans on both types of static stimuli, thereby extending previous findings on target emotion recognition (Krumhuber et al., 2021a). With the absence of video ratings from human observers, however, no firm conclusion can be drawn regarding the role of movement versus static information in human emotion classification. To rectify this shortcoming, a second study was conducted in which human observers rated all three types of stimuli: video (dynamic), target and non-target images (static).

## 3. Experiment 2

The second study aimed to replicate and extend the findings of the first study with solely human observers, thereby using a subset of the stimuli and a larger sample of participants. For this purpose, we obtained human ratings of three stimulus types (video, target, and non-target images) and analysed the relative contribution of prototypicality, ambiguity, and complexity to emotion classification. We further explored the extent to which video recognition can be predicted based on performance for single images.

### 3.1. Method

#### 3.1.1. Stimulus material

To select a diverse set of stimuli, 8 videos per emotion were taken from Study 1. This resulted in a total of 48 videos (8 videos × 6 emotions) and 288 images (48 videos × 6 images). The size of the image and video stimuli was approximately 550 × 440 pixels.

#### 3.1.2. Human observers

##### 3.1.2.1. Participants

Three hundred and three participants (141 females), aged between 18–60 years (*M* = 35.99, *SD* = 10.84), volunteered to take part in the study. Participants were recruited online via a digital recruitment platform (Academic Prolific). Participants were compensated £7 for taking part in the study. All participants were White/Caucasian who identified themselves as British or European and were ordinary residents in the UK. Ethical approval was granted by the Department of Experimental Psychology at University College London, United Kingdom.

##### 3.1.2.2. Procedure

The experiment was programmed using the Qualtrics software (Provo, UT). In the first block, participants were randomly presented with one of the six images extracted from each video, yielding 48 images showing each of the six basic emotions. In the second block, 48 videos displaying each of the six basic emotions in dynamic form were presented in a randomized order. Measures of emotion recognition were the same as in Study 1.

### 3.2. Results

#### 3.2.1. 6-images as predictor of video recognition

We first tested whether the 6 images can predict how well the video is recognised. For this, a multilevel logistic regression model predicting video-level emotion classification accuracy (by human) was estimated with a random intercept for each video and fixed slope for the sum of correct image-level emotion classification accuracy (per video). The results revealed a significant main effect (exp(β) = 2.43, Wald = 11.99, *p* < 0.001, exp(95% CI) [1.47, 4.03]), indicating that the odds of correct video emotion classification increased by143% for each additional correctly classified image.

#### 3.2.2. Video vs. target image vs. non-target images

**Figure 4:**
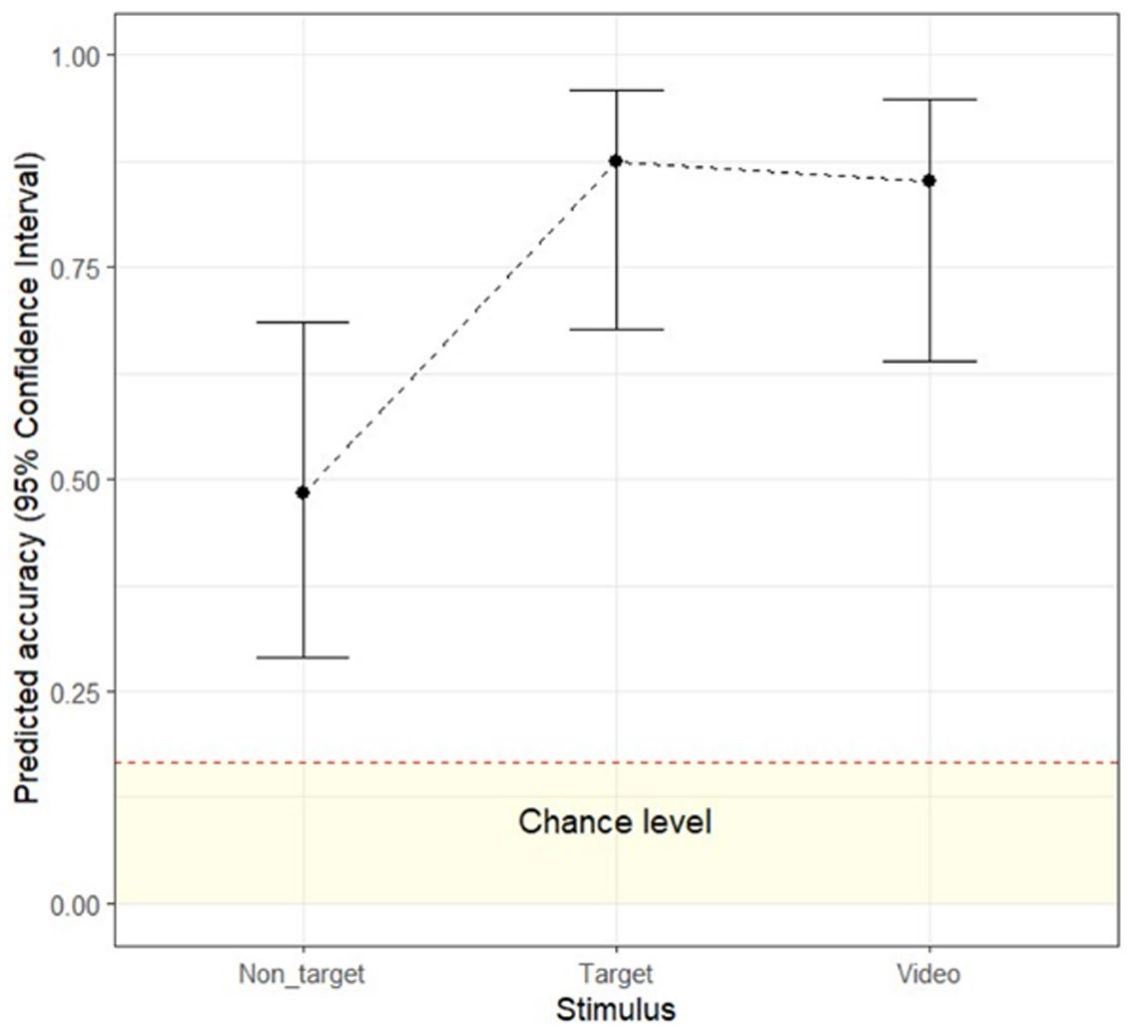
Human recognition accuracy for video, target- and non-target images. Error bars represent upper and lower 95% confidence interval. Dashed red line indicates 1/6 conservative chance level (Krumhuber et al., 2021b).

To examine whether recognition accuracy differs as a function of stimulus type (video vs. target image vs. non-target images), a multilevel logistic regression analysis with a random intercept by video was conducted on the human accuracy data. The odds of correct emotion classification were significantly higher for target images than for non-target images (exp(β) = 7.43, Wald = 15.29, *p* < 0.001, exp(95%CI) [2.72, 20.32]) and were significantly higher for the video (exp(β) = 6.11, Wald = 13.16, *p* < 0.001, exp(95%CI) [2.30, 16.26]) than for non-target images. The odds of correct emotion classification were not significantly different between the target image and the video (exp(β) = 0.82, Wald = 0.10, *p* = 0.947, exp(95%CI) [0.24, 2.80]). Similar to Study 1, the dynamic advantage only occurred when the video was compared to non-target images, but not target images (see Figure 4).

#### 3.2.3. Prototypicality, ambiguity, and complexity of expression

Using the machine data, we assessed prototypicality, ambiguity, and complexity of the stimulus types (target and non-target images). Overall, Welch’s *t*-tests showed that target images were significantly more prototypical (*M_target_* = 80.82, *SD* = 27.17 vs. *M_non-target_* = 56.36, *SD* = 32.84), *t*(77.18) = 5.49, *p* < 0.001, *d* = 0.76, less ambiguous (*M_target_* = 14.53, *SD* = 14.25 vs. *M_non-target_* = 33.71, *SD* = 21.22), *t*(94.25) = −7.76, *p* < 0.001, *d* = 0.95, and more complex (*M_target_* = 27.38, *SD* = 6.73 vs. *M_non-target_* = 22.36, *SD* = 8.96), *t*(84.17) = 4.44, *p* < 0.001, *d* = 0.58 than non-target images. As such, the subset of 48 stimuli was sufficiently representative of the larger sample analysed in Study 1.

Next, we examined the partial contribution of each parameter to human emotion classification accuracy of images. For this, a multilevel logistic regression model predicting each image’s classification accuracy was estimated with random intercepts for each video and fixed slopes for prototypicality, ambiguity, and complexity. Results revealed a significant main effect of ambiguity (exp(β) = 0.96, Wald = 7.60, *p* = 0.006, exp(95%CI) [0.94, 0.99]), complexity (exp(β) = 1.16, Wald = 13.63, *p* < 0.001, exp(95%CI) [1.07, 1.25]), and a marginally significant effect of prototypicality (exp(β) = 1.01, Wald = 3.14, *p* = 0.076, exp(95%CI) [1.00, 1.03]). In general, the odds of recognition accuracy increased by 1 and 16% for a unit increase in prototypicality and complexity respectively, while they decreased by 4% for a unit increase in ambiguity (see Table 3).

**Table 3:** Model estimates for FACET image recognition accuracy, showing main effect estimates in logits, upper and lower bounds of exponentiated 95% confidence intervals, and significance of each predictor (Study 1).

Predictor	exp(β)	Wald	L95%CI	H95%CI	*p*
Prototypicality	1.01	3.14	1.00	1.03	0.076
Ambiguity	0.96	7.60	0.94	0.99	0.006**
Complexity	1.16	13.63	1.07	1.25	>0.001***

Finally, we explored the predictive power of each parameter for human video recognition. For this, a multilevel logistic regression model predicting human video-level emotion classification accuracy was developed with random intercepts for each source database and fixed slopes for video-level prototypicality, ambiguity, and complexity. The results revealed a significant main effect of ambiguity (exp(β) = 0.95, Wald = 5.04, *p* = 0.025, exp(95%CI) [0.90, 0.99]), indicating that the odds of recognition accuracy decreased by 5% for each unit increase in ambiguity. The main effects of prototypicality (exp(β) = 0.99, Wald = 0.23, *p* = 0.629, exp(95%CI) [0.96, 1.02]) and complexity (exp(β) = 1.06, Wald = 1.23, *p* = 0.267, exp(95%CI) [0.96, 1.22]) were not significant (see Table 4).

**Table 4:** Model estimates for human video recognition accuracy, showing main effect estimates in logits, upper and lower bounds of exponentiated 95% confidence intervals, and significance of each predictor (Study 2).

Predictor	exp(β)	Wald	L95%CI	H95%CI	*p*
Prototypicality	0.99	0.23	0.96	1.02	0.629
Ambiguity	0.95	5.04	0.90	0.99	0.025*
Complexity	1.06	1.23	0.96	1.22	0.267

### 3.3. Discussion

Similar to the first study, there were substantial differences in emotion recognition accuracy across stimulus types. While target images and videos were similarly well recognised, accuracy for non-target images was significantly reduced. As such, movement may function as a facilitative factor particularly when static information fails to convey the target peak emotion. Correct classification of the extracted images was predictive of human recognition performance for the full video, suggesting that single images may be useful for conveying a given expression. As in Study 1, higher complexity but lower ambiguity contributed to classification accuracy. Furthermore, the effect of prototypicality was only marginally significant, with facial expressions likely to be processed by humans more holistically and in an integrated fashion (Calder et al., 2000b; Calvo et al., 2012). Together, these findings suggest that categorical ambiguity and complexity (overall expressivity) play an important role in human emotion recognition which seems to rely on features other than prototypicality.

## 4. General discussion

Past research has been inconclusive with regards to the conditions in which dynamic information matters. In two studies, dynamic expressions were more accurately classified than non-target images, with temporal information aiding emotion recognition. The results partially replicate previous findings on the dynamic advantage (Ambadar et al., 2005; Bould and Morris, 2008; Cassidy et al., 2015), showing that facial expressions are temporally structured in a way that is both meaningful and beneficial to observers. However, these movement-related benefits disappeared in comparison to static peak expressions of the target emotion. Insofar as target images represented static snapshots of a fully expressed emotion, they may have provided sufficient information for emotion classification. This was not the case for non-target images captured at various time points and indicative of peak expressions other than the target emotion. Together, these findings suggest a compensatory role of dynamic information, facilitating emotion recognition when static emotional cues are suboptimal or insufficient (Ehrlich et al., 2000; Wehrle et al., 2000; Atkinson et al., 2004).

In support of this notion, non-target images were found to be less prototypical and complex, as well as more ambiguous. Similar to past research (Matsumoto et al., 2009; Matsumoto and Hwang, 2014) prototypicality played a crucial role, with expressions that more closely resemble BET predictions (Ekman et al., 2002) enhancing recognition. This applied particularly to the machine due to its history of training on posed/stylised expressions. For human observers, complexity was more important for emotion recognition. Consistent with previous work (Matsumoto et al., 2002; Jones et al., 2018), expression intensity (as measured by our new complexity metric) notably improved performance. Here, we showed for the first time that complexity can explain recognition performance without having to confound intensity with prototypicality and its BET-based assumptions. In the future, this allows for subtle expressions to be coded separately from non-prototypical expressions as both metrics tap into different characteristics. As predicted, ambiguous expressions were often subject to misclassification, with the simultaneous presentation of contradictory emotional cues increasing human and machine difficulty in recognising discrete emotions (Calder et al., 2000b; Neta and Whalen, 2010). While previous studies mainly relied on techniques to create ambiguous stimuli, the present research introduced a new metric for *quantifying* ambiguity. This metric can be applied to any emotion rating data in future research that provides a probability for a closed set of emotion categories.

Machine recognition exceeded human performance for both types of static images. The finding extends prior work (Krumhuber et al., 2021a,b) by demonstrating a machine advantage for classifying expressions at the peak of the target emotion as well as other time points of the facial display (non-target images). In contrast to earlier studies showing a reduction in machine performance for low-intensity expressions (Calvo et al., 2018; Küntzler et al., 2021), we found that non-target images were better recognised by the machine than human observers despite their substantially lower prototypicality, greater ambiguity, and lower complexity. It should be noted, however, that stimuli were drawn from standardised datasets, which may benefit machine analysis (Pantic and Bartlett, 2007). Furthermore, our extraction procedure was designed to select peak images for other emotions to examine the underlying featural parameters. Therefore, the non-target images primarily differed from the target images in ambiguity and prototypicality, and less in complexity or intensity. Here, future work could systematically manipulate all three parameters to better understand their impact on human and machine recognition performance.

There is no doubt that video rating studies are costly and resource intensive. Automatic peak extraction may be an economic choice for addressing certain research questions by reducing the required presentation time of each stimulus. After accounting for potential fatigue effects in our human sample, we could present three times as many image stimuli in Study 1 than video stimuli in Study 2. This was the case even though our videos were relatively short and standardised. As is now widely recognised in the field, there is a need for studying more ecological behaviours such as those observed in the wild (Krumhuber et al., 2017; Küster et al., 2020, 2022). However, naturalistic stimuli tend to be considerably longer, less standardised, and less well annotated (Cowie et al., 2005; Girard et al., 2015; Benitez-Quiroz et al., 2016). Here, algorithmic approaches could help by allowing thin slices of stimulus materials to be presented to participants. These could be static peak images or frame sequences extracted on the basis of machine parameters. As such, AFEA may provide a valuable tool to systematically define and extract appropriate research materials from otherwise seemingly “unwieldy” naturalistic datasets.

While present methods for identifying peak images vary between studies (Stöckli et al., 2018; Skiendziel et al., 2019; Onal Ertugrul et al., 2023), both expert-based and algorithmic selection may be subject to biases (e.g., human experts might discard images that appear too ambiguous due to the presence of additional action units). Here, an algorithmic may be more objective because each action unit is assessed separately. However, algorithmic peak selection may suffer from other types of biases. For example, variable lighting during a video might result in the machine missing certain peaks that a trained human expert could have recognised. Thus, although algorithmic approaches might be particularly helpful for studying naturalistic datasets, further research will still be required to assess the reliability of these tools for more “in the wild” recordings.

The present work has taken first steps to blend AFEA with psychological research on human emotion recognition. The results extend previous work by introducing complexity as a novel metric of intensity that is largely decoupled from prototypicality and BET. We argue that featural parameters such as prototypicality, ambiguity, and complexity reveal important new insights into human vs. machine differences. Specifically, complexity is a defining feature for humans who are likely to process expressions in a more integrated fashion. In contrast, machine algorithms such as FACET still mainly rely on prototypicality, achieving better performance on peak images than videos, especially if those are highly prototypical and complex, and low in ambiguity. The present research helps inform psychological studies into the mechanisms that underlie the dynamic advantage. Closing this knowledge might be particularly fruitful for future work on dynamic spontaneous expressions.^1^

## Data availability statement

The raw data supporting the conclusions of this article will be made available by the authors, without undue reservation.

## Ethics statement

The studies involving humans were approved by Department of Psychology, University College London. The studies were conducted in accordance with the local legislation and institutional requirements. The participants provided their written informed consent to participate in this study. Written informed consent was obtained from the individual(s) for the publication of any potentially identifiable images or data included in this article.

## Author contributions

EK, DK, and HK conceived and designed the experiments. HK performed the experiments and wrote the first draft of the manuscript. HK conducted the statistical analysis under the guidance of JG. JG formalized the statistical definitions of the feature parameters. HK, DK, JG, and EK reviewed and/or edited the manuscript before submission. All authors contributed to the article and approved the submitted version.

## Conflict of interest

The authors declare that the research was conducted in the absence of any commercial or financial relationships that could be construed as a potential conflict of interest.

## Publisher’s note

All claims expressed in this article are solely those of the authors and do not necessarily represent those of their affiliated organizations, or those of the publisher, the editors and the reviewers. Any product that may be evaluated in this article, or claim that may be made by its manufacturer, is not guaranteed or endorsed by the publisher.

## Footnotes

^1^Random effect variables (database and video) slightly differed for predicting video and image recognition accuracy. These random effects were selected by comparing the likelihood ratio of fit models containing different random effect variables. A model showed a boundary fit for minimal amounts of random effect observed, however results remained the same with and without the random effects.
